# Metabolomic Profile of Weaned Pigs Challenged with *E. coli* and Supplemented with Carbadox or *Bacillus subtilis*

**DOI:** 10.3390/metabo11020081

**Published:** 2021-01-30

**Authors:** Yijie He, Yanhong Liu, Peng Ji

**Affiliations:** 1 Department of Animal Science, University of California, Davis, CA 95616, USA; yjhe@ucdavis.edu; 2 Department of Nutrition, University of California, Davis, CA 95616, USA; penji@ucdavis.edu

**Keywords:** *Bacillus subtilis*, carbadox, *Escherichia coli*, intestine, metabolomics, weaned pigs

## Abstract

This study explored the metabolomic profiles in ileal mucosa and colon digesta in response to enterotoxigenic *Escherichia coli* F18 (ETEC) infection and dietary use of probiotics and low-dose antibiotics. Weaned pigs (*n* = 48, 6.17 ± 0.36 kg body weight) were randomly allotted to one of four treatments. Pigs in the negative control (NC) were fed a basal diet without ETEC challenge, whereas pigs in the positive control (PC), antibiotic, and probiotic groups were fed the basal diet, basal diet supplemented with 50 mg/kg of carbadox, or 500 mg/kg of *Bacillus subtilis*, respectively, and orally challenged with ETEC F18. All pigs were euthanized at day 21 post-inoculation to collect ileal mucosa and colon digesta for untargeted metabolomic profiling using gas chromatography coupled with time-of-flight mass spectrometry. Multivariate analysis highlighted a more distinct metabolomic profile of ileal mucosa metabolites in NC compared to the ETEC-challenged groups. The relative abundance of 19 metabolites from the ileal mucosa including polyamine, nucleotide, monosaccharides, fatty acids, and organic acids was significantly different between the NC and PC groups (*q* < 0.1). In colon digesta, differential metabolites including 2-monoolein, lactic acid, and maltose were reduced in the carbadox group compared with the probiotics group. In conclusion, several differential metabolites and metabolic pathways were identified in ileal mucosa, which may suggest an ongoing intestinal mucosal repair in the ileum of ETEC-challenged pigs on day 21 post-inoculation.

## 1. Introduction

In the swine industry, enterotoxigenic *E. coli* (ETEC) induced enteric infection is one of the leading causes of morbidity in weanling pigs [1]. The diarrheal disease caused by ETEC results in significant economic losses due to the high rate of morbidity and mortality if pigs are left untreated, and increased cost of production due to medical treatment, vaccination, and feed supplements [2]. According to surveys conducted by the United States Department of Agriculture (USDA) national animal health monitoring systems (NAHMS), from the years 2000 to 2012, *E. coli* diarrhea was reported to have affected 32.1%, 45.5%, and 32.4% the medium-scale farms (2000 to 4999 heads), respectively. From 1995 to 2012, mortality rates of nursery pigs ranged from 2.4 to 3.6%, of which diarrhea-caused deaths were from 9.4 to 14.7% [3]. Extensive research has been done to investigate the specific mechanisms of ETEC post-weaning diarrhea. It is now understood that the pathogenesis of ETEC post-weaning diarrhea primarily involves two steps including the attachment of the bacteria to the enterocytes of the small intestine, and the subsequent production of enterotoxins that induces secretory diarrhea, dehydration, and emaciation of pigs [1]. ETEC infection reduces the growth performance of pigs, reduces intestinal villi height, and increases the transcellular and paracellular permeability of the jejunum [4,5]. Moreover, it can elicit local and systemic immune responses such as the production of proinflammatory cytokines and recruitment of immune cells [6,7]. 

The molecular mechanism of host responses to ETEC infection has also been recently investigated in several studies with emerging systemic approaches such as transcriptomic, proteomic, and metabolomics analysis [8,9,10]. For instance, Zhou et al. [8] reported that ETEC infection greatly upregulated the expression of genes related to immune response and cell cycle progression. Wu et al. [10] identified a wide range of differential metabolites in the jejunum that are involved in energy metabolism and are a protective mechanism against ETEC diarrhea. However, little is known regarding the metabolic changes in the ileum and colon of ETEC-challenged pigs. Moreover, ETEC challenge protocols in weaned pigs have been developed and applied widely in different studies to evaluate the efficacy of feed additives in nursery diet as alternatives to antibiotics [5,11,12,13]. It is of interest to investigate the molecular mechanisms of these feed additives in alleviating ETEC post-weaning diarrhea. Therefore, the primary objective of the present study was to explore the differences in the metabolic profiles in the ileal mucosa and colon digesta between enterotoxigenic *Escherichia coli* F18-challenged and non-challenged pigs as well as in pigs supplemented with carbadox or a *Bacillus subtilis* probiotic.

## 2. Results

### 2.1. Metabolite Profile in Ileal Mucosa

**Figure 1:**
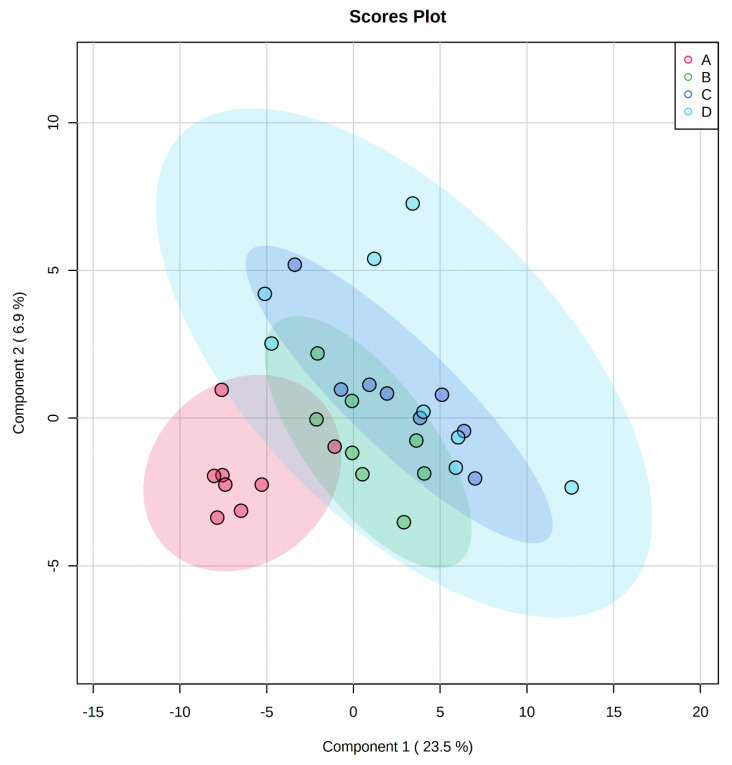
Partial Least Squares Discriminant Analysis (PLS-DA) 2D score plot of the metabolites in ileal mucosa showed separated clusters between the negative control and positive control groups. 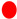, negative control (**A**), 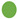, positive control (**B**), 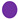, carbadox (**C**), and 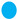, probiotic (**D**). Shaded areas in different colors represent the 95% confidence interval.

**Figure 2:**
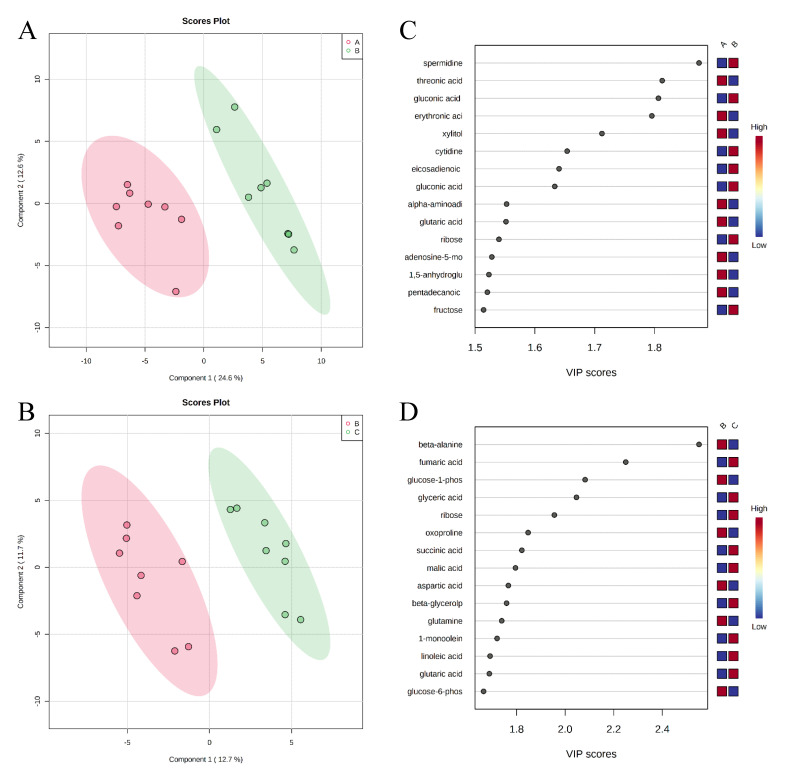
Partial Least Squares Discriminant Analysis (PLS-DA) score plots of metabolites in ileal mucosa (**A**,**B**) showed a clear separation between the negative control (**A**) and positive control (**B**) groups, and positive control (**B**) and carbadox (**C**) groups, respectively. The top 15 metabolites with VIP > 1 scores are shown in (**C**,**D**).

**Figure 3:**
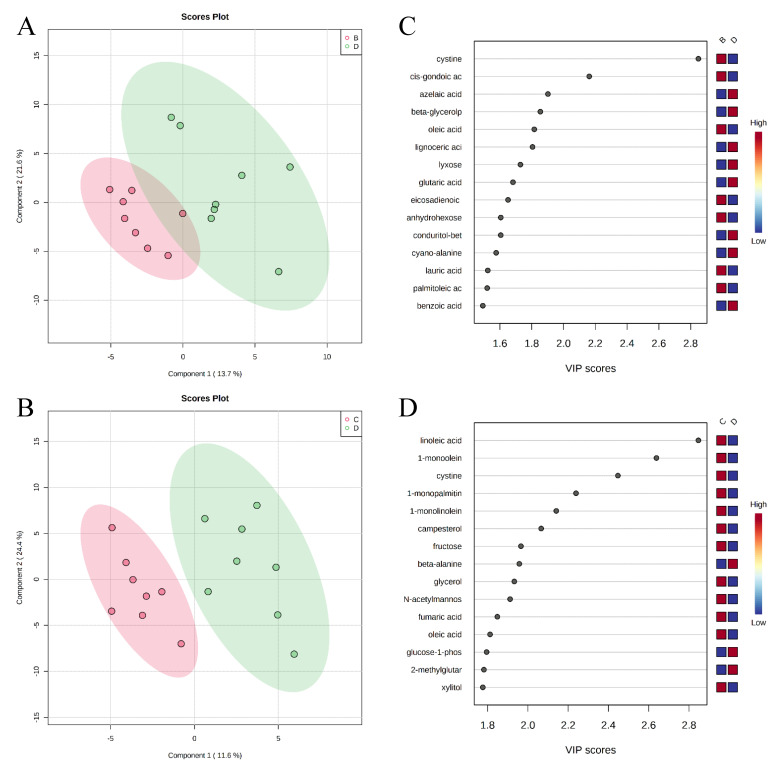
Partial Least Squares Discriminant Analysis (PLS-DA) score plots of metabolites in ileal mucosa (**A**,**B**) showed clear separation between positive control (**B**) and probiotic (**D**) groups, and carbadox (**C**) and probiotic (**D**) groups, respectively. The top 15 metabolites with VIP > 1 scores are shown in (**C**,**D**).

A total of 350 metabolites (141 identified and 209 unidentified) were detected in ileal mucosa. Based on the identified metabolites, a Partial Least Squares Discriminant Analysis (PLS-DA) score plot with 95% confidence ranges (circled areas) showed clear separation between the negative control and positive control groups (Figure 1). To further explore the metabolic profile differences among the four dietary treatments, PLS-DA was performed for the following comparisons: (1) negative control vs. positive control, (2) positive control vs. carbadox, (3) positive control vs. probiotics, and (4) carbadox vs. probiotics. The score plot of PLS-DA again distinguished the negative control from the positive control (Figure 2A), and the top 15 metabolites identified by Variable Importance in Projection (VIP) score are shown in Figure 2C. The score plots of PLS-DA also distinguished the positive control from the carbadox groups (Figure 2B), the positive control and probiotics groups (Figure 3A), and the carbadox and probiotics groups (Figure 3B), with the top 15 metabolites with the highest VIP scores for each comparison shown in Figure 2D, Figure 3C, and Figure 3D, respectively. In comparison to the negative control, 11 metabolites (spermidine, cytidine, gluconic acid, gluconic acid lactone, gulonic acid, fructose, fructose-6-phosphate, mannose, sorbitol-6-phosphate, glucose, and glucose-6-phosphate) were increased, while eight metabolites (adipic acid, pentadecanoic acid, lignoceric acid, glutaric acid, pyrophosphate, conduritol-beta-epoxide, adenosine-5-monophosphate, and succinic acid) were decreased in the positive control (Table 1). Pigs in the carbadox group had less 1-monoolein in the ileal mucosa than pigs in the probiotics group. However, no deferential metabolites were identified when we compared the positive control vs. carbadox groups and the positive control vs. probiotics groups. 

**Figure 4:**
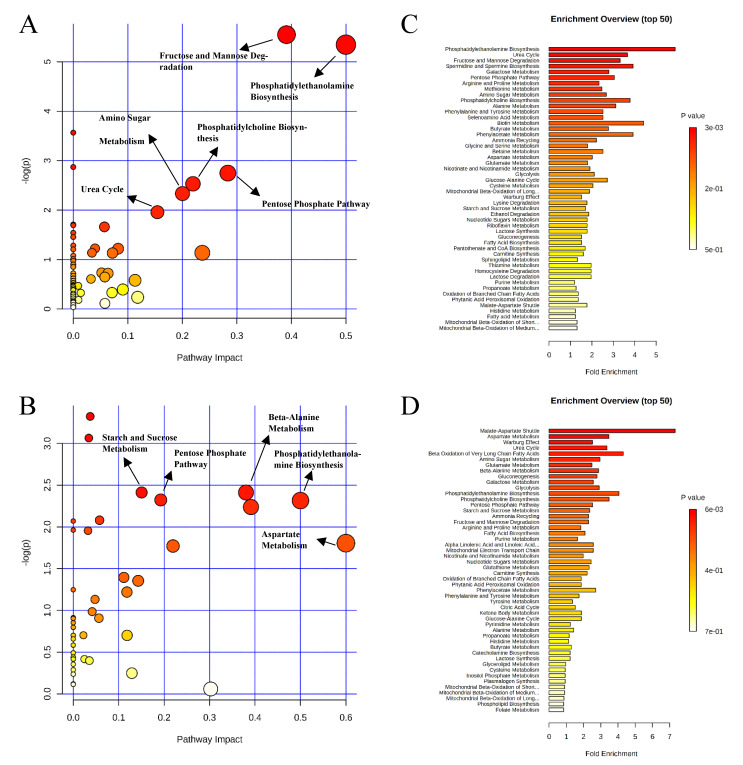
Significantly changed pathways in the ileal mucosa between the negative control and positive control groups (**A**), and positive control and carbadox groups (**B**). The x-axis represents the pathway impact values and the y-axis represents the −log *p*-values from the pathway enrichment analysis. Metabolite set enrichment analysis (**C**,**D**) shows the metabolic pathways were enriched in the negative control compared to the positive control, and the positive control and carbadox, respectively. Both pathway analysis and metabolite set enrichment analysis were performed using identified metabolites with VIP > 1.

**Figure 5:**
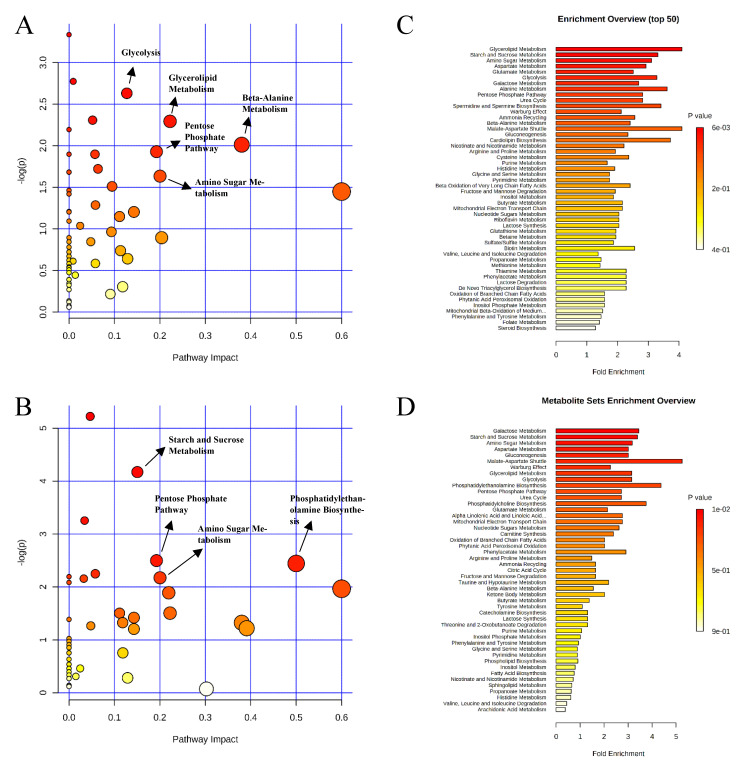
Significantly changed pathways in the ileal mucosa between the positive control and probiotic groups (**A**), and carbadox and probiotic groups (**B**). The x-axis represents the pathway impact values and the y-axis represents the −log *p*-values from pathway enrichment analysis. Metabolite set enrichment analysis (**C**,**D**) shows metabolic pathways enriched in the positive control compared to the probiotic, and carbadox and the probiotic, respectively. Both pathway analysis and metabolite set enrichment analysis were performed using identified metabolites with VIP > 1.

Pathway analysis and metabolite set enrichment analysis were performed on metabolites with VIP > 1. Phosphatidylethanolamine biosynthesis, fructose and mannose degradation, pentose phosphate pathway, phosphatidylcholine biosynthesis, amino sugar metabolism, and urea cycle were the most affected metabolic pathways (−log *p* value > 2) when comparing the positive control with the negative control (Figure 4A,C). Phosphatidylethanolamine biosynthesis, beta-alanine metabolism, pentose phosphate pathway, starch and sucrose metabolism, and fructose and mannose degradation were the most affected metabolic pathways when comparing the positive control with the carbadox group (Figure 4B,D). Beta-alanine metabolism, glycerolipid metabolism, glycolysis, pentose phosphate pathway, and amino sugar metabolism were the most affected metabolic pathways when the positive control was compared with the probiotics group (Figure 5A,C). However, phosphatidylethanolamine biosynthesis, amino sugar metabolism, the pentose phosphate pathway, and starch and sucrose metabolism were the most affected metabolic pathways in the comparison of the carbadox vs. probiotics group (Figure 5B,D). 

### 2.2. Metabolite Profile in Colon Digesta

**Figure 6:**
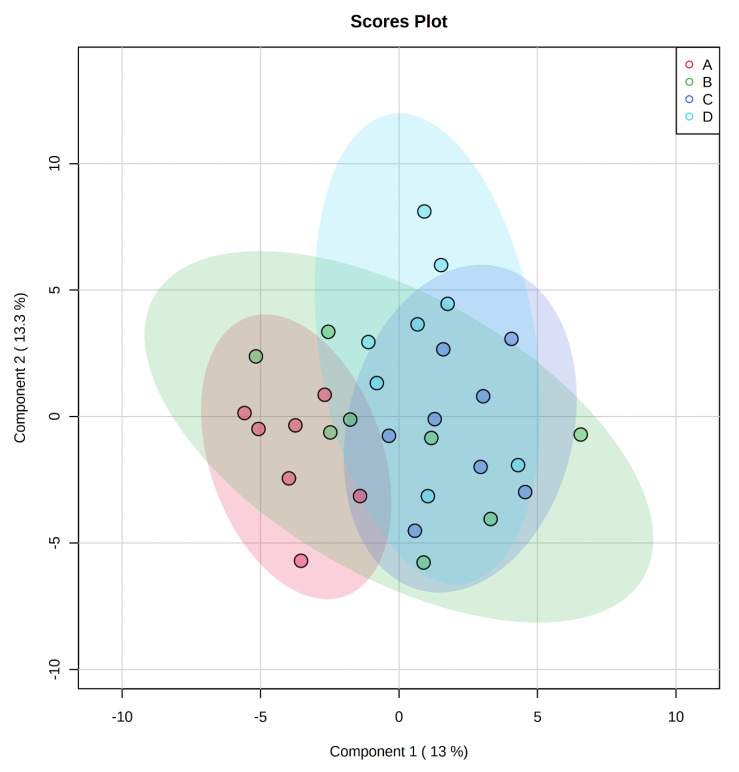
Partial Least Squares Discriminant Analysis (PLS-DA) 2D score plot of metabolites in colonic mucosa. No clear separation of clusters was observed. 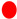, negative control (**A**), 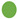, positive control (**B**), 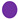, carbadox (**C**), and 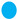, probiotic (**D**). Shaded areas in different colors represent the 95% confidence interval.

**Figure 7:**
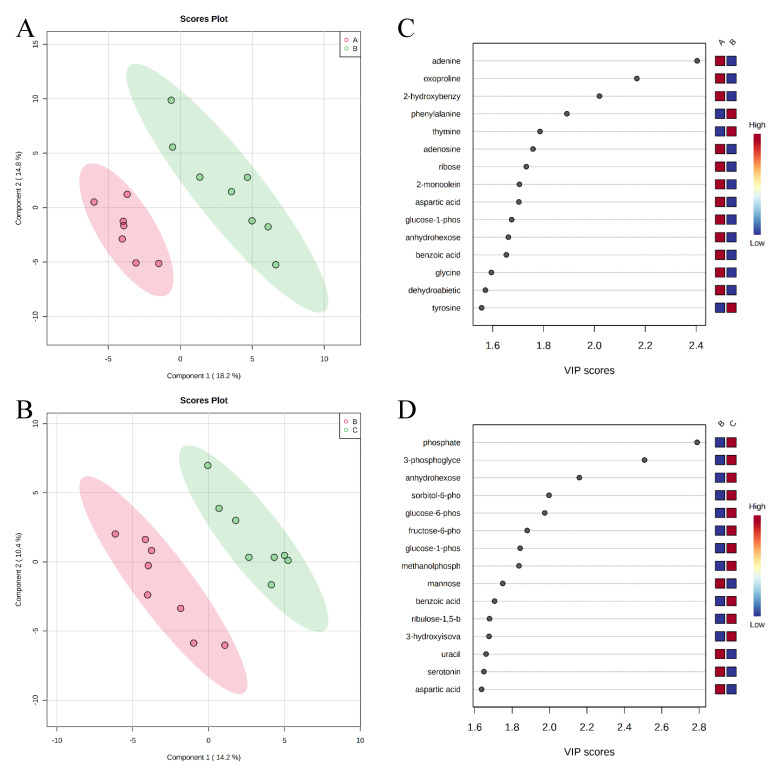
Partial Least Squares Discriminant Analysis (PLS-DA) score plots of metabolites in colonic mucosa (**A**,**B**) showed clear separation between the negative control (**A**) and positive control (**B**) groups, and positive control (**B**) and carbadox (**C**) groups, respectively. The top 15 metabolites with VIP > 1 scores are shown in (**C**,**D**).

**Figure 8:**
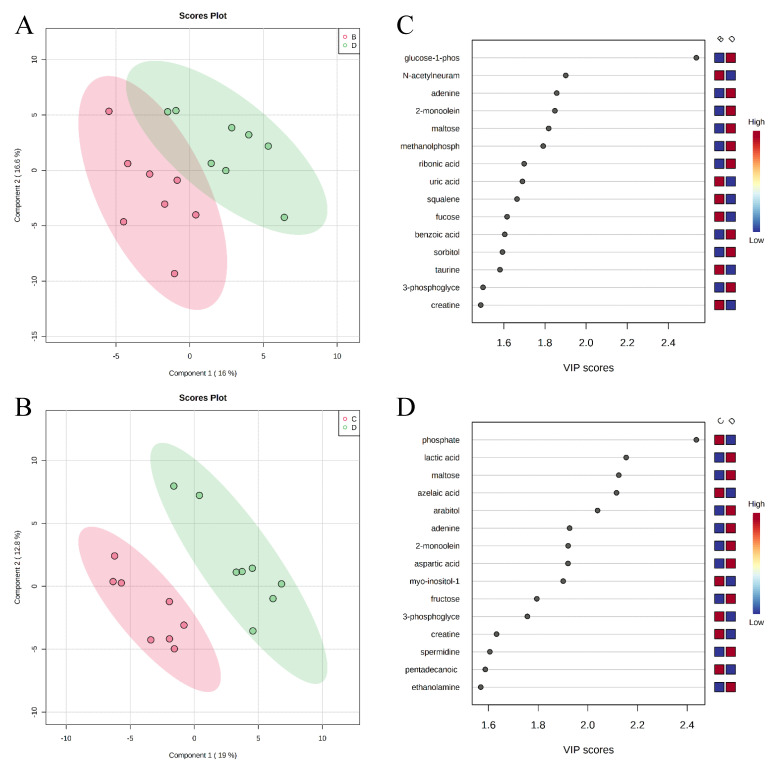
Partial Least Squares Discriminant Analysis (PLS-DA) score plots of metabolites in colonic mucosa (**A**,**B**) showed clear separation between the positive control (**B**) and probiotic (**D**) groups, and the carbadox (**C**) and probiotic (**D**) groups, respectively. The top 15 metabolites identified with VIP > 1 scores are shown in (**C**,**D**).

A total of 284 metabolites (128 identified and 156 unidentified) were detected in colon digesta. Based on the identified metabolites, a PLS-DA score plot showed overlaps among treatment groups (Figure 6). When considered in a pairwise manner, the score plot of PLS-DA distinguished the negative control and positive control groups (Figure 7A), and the top 15 metabolites identified by VIP score are shown in Figure 7C. The score plots of PLS-DA also distinguished the positive control vs. carbadox (Figure 7B), positive control vs. probiotics (Figure 8A), and carbadox vs. probiotics (Figure 8B), with the top 15 metabolite features shown in Figure 7D, Figure 8C, and Figure 8D, respectively. Adenine was reduced in the colon digesta of pigs in the positive control compared with pigs in the negative control (Table 2). Phosphate was enriched in the colon digesta of pigs in the carbadox group compared with pigs in the positive control. Five metabolites (2-monoolein, lactic acid, maltose, adenine, aspartic acid) were reduced, while phosphate was enriched in the carbadox group compared with the probiotics group. No differential metabolites were identified between the positive control and probiotics groups.

**Figure 9:**
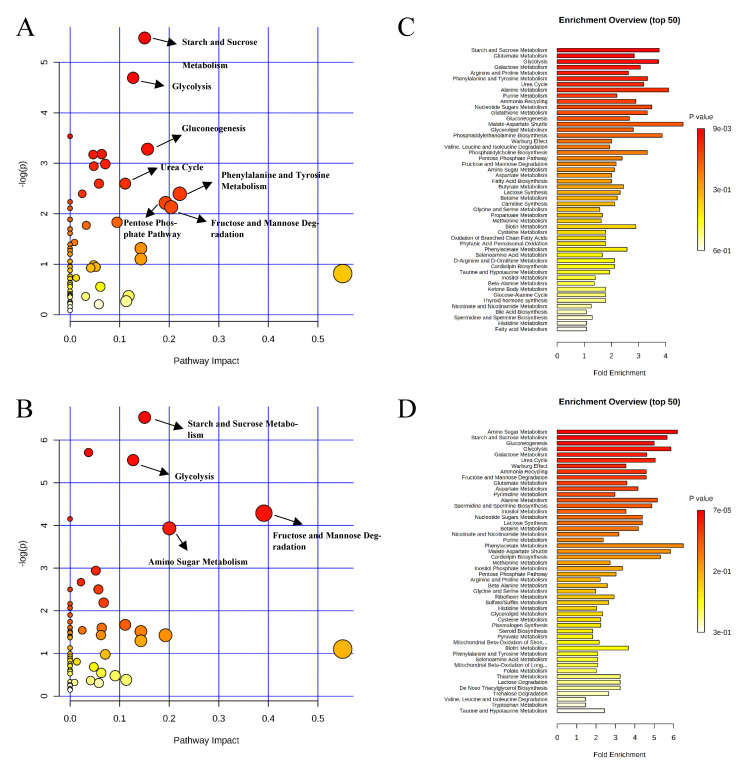
Significantly changed pathways in the colon digesta between the negative control and positive control groups (**A**), and positive control and carbadox groups (**B**). The x-axis represents the pathway impact values and the y-axis represents the −log *p*-values from pathway enrichment analysis. Metabolite set enrichment analysis (**C**,**D**) shows metabolic pathways enriched in the negative control compared to the positive control, and the positive control and carbadox, respectively. Both pathway analysis and metabolite set enrichment analysis were performed using identified metabolites with VIP > 1.

**Figure 10:**
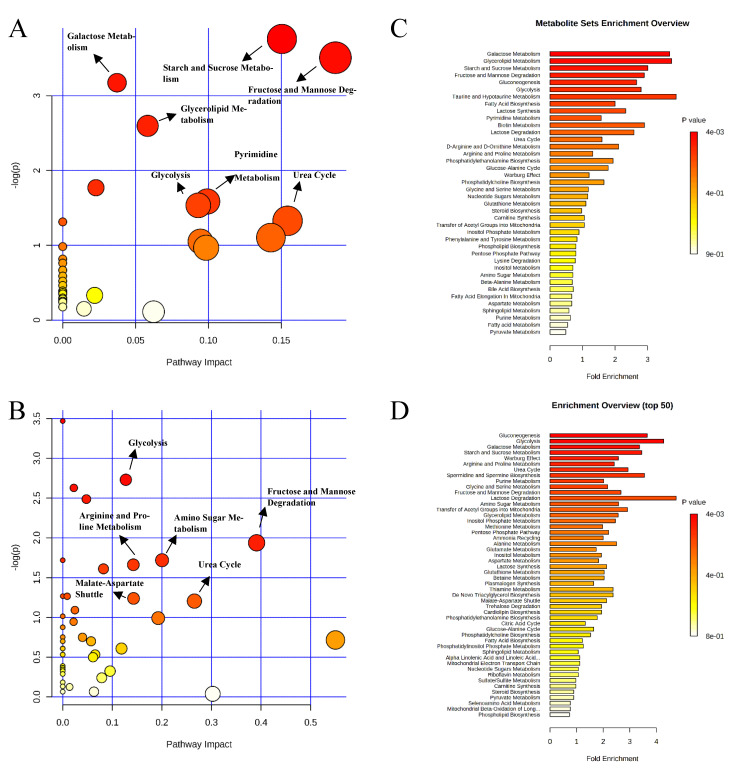
Significantly changed pathways in the colon digesta between the positive control and probiotic groups (**A**), and carbadox and probiotic groups (**B**). The x-axis represents the pathway impact values and the y-axis represents the −log *p*-values from pathway enrichment analysis. Metabolite set enrichment analysis (**C**,**D**) shows that the metabolic pathways were enriched in the positive control compared to the probiotics, and carbadox and probiotics, respectively. Both pathway analysis and metabolite set enrichment analysis were performed using identified metabolites with VIP > 1.

Pathway analysis and metabolite set enrichment analysis were performed on metabolites in colon digesta with VIP > 1. Starch and sucrose metabolism, glycolysis, gluconeogenesis, urea cycle, phenylalanine and tyrosine metabolism, fructose and mannose degradation, and the pentose phosphate pathway were the most affected metabolic pathways when comparing the positive control with the negative control (Figure 9A,C). Starch and sucrose metabolism, glycolysis, fructose and mannose degradation, and amino sugar metabolism were the most affected metabolic pathways when comparing the carbadox group with the positive control (Figure 9B,D). In a comparison of the positive control with the probiotics group, fructose and mannose degradation, starch and sucrose metabolism, galactose metabolism, urea cycle, pyrimidine metabolism, glycolysis, and glycerolipid metabolism were the most affected metabolic pathways (Figure 10A,C). Fructose and mannose degradation, amino sugar metabolism, arginine and proline metabolism, glycolysis, urea cycle, and malate-aspartate shuttle were the most affected metabolic pathways when the probiotics group was compared with the carbadox group (Figure 10B,D).

## 3. Discussion

Growing evidence indicates that ETEC infection can inhibit pigs’ intestinal immune responses and induce profound changes to many metabolic processes in the jejunum [8,9,14]. Little is known about the metabolic changes in the ileum of ETEC challenged pigs, although ETEC generally proliferate and colonize from the mid-jejunum to ileum [2]. In the present study, the metabolite profile of ileum mucosa and colon digesta of weaned pigs challenged with an ETEC F18 were investigated using an untargeted metabolomics approach. Nineteen identified metabolites were altered by infection at day 21 post-inoculation (PI). Data in growth performance, clinical signs, systemic inflammation, and intestinal morphology have already been reported in our previous research [5]. Pigs challenged with ETEC F18 recovered from the infection as indicated by no diarrhea symptoms and comparable intestinal morphology compared with pigs in the negative control. Results from the current study suggest a prolonged effect of ETEC F18 infection on ileal mucosal metabolism. These metabolites can be categorized into polyamine, nucleotide, monosaccharides, fatty acids, and organic acids. In contrast, ETEC infection did not affect colonic metabolism at d 21 PI.

### 3.1. Metabolites Related to Host Metabolism

Previous challenge studies in weaned pigs have demonstrated the immediate and short-term negative effects of an ETEC challenge on pigs’ intestinal health and growth performance including watery diarrhea, activated intestinal inflammation response, increased intestinal permeability, disrupted gut microbiota homeostasis, and decreased average daily gain [4,5,12,15]. It should be noted that the current study investigated the metabolic profiles of ileum mucosa and colon digesta at d 21 PI, when pigs were cleared of ETEC F18. Previous studies have shown that the reduction in watery diarrhea occurred around d 5 PI and the causative beta-hemolytic ETEC F18 was cleared between d 9 and d 11 PI [4,5,11]. However, pigs may still experience systemic and local inflammation on d 11 PI, as indicated by a high number of neutrophils in blood, high concentration of haptoglobin in serum, and increased expression of inflammatory mediators in the ileal mucosa of ETEC-infected pigs compared with pigs from sham control groups [4,12,16].

In the present study, differences in the metabolic profiles between ETEC-challenged and non-challenged pigs were found mostly in the ileal mucosa. This finding suggests that ETEC infection induced metabolic changes in the ileum, and supports the site of pathogenesis of ETEC as it can proliferate and colonize the ileum of pigs [2]. A PLS-DA score plot constructed based on the metabolite profiles of the ileal mucosa showed a clear separation between ETEC-challenged and non-challenged pigs, regardless of dietary interventions (Figure 1). These findings suggest that the changes in ileal mucosal metabolism induced by the ETEC infection were not restored by the dietary supplementation of carbadox or *Bacillus subtilis*. In principle, carbadox interferes with bacterial DNA synthesis, causing the breakdown of chromosomes in *E. coli* [17,18]. *Bacillus subtilis* exerts beneficial effects through modulation of host immune responses, enhancement of the expression of tight junction proteins, and production of antimicrobials [19,20,21]. The lack of significant treatment effect on ileal metabolic profiles in the present study may be that the ETEC infection mainly induced cellular activities such as host cell-cycle progress and immune response, but carbadox or the *Bacillus subtilis* probiotic may only exert their effects from the aspect of immune response [8]. 

It was previously reported that ETEC F18 impaired epithelial integrity in the small intestine [4,5,12] and caused villus shortening, epithelial cell wasting and apoptosis, and increased number of sloughed epithelial cells [22,23,24]. Mucosal repair processes were shown to be activated in response to infection and reinstate epithelial barrier functions [25]. Spermidine is an important polyamine that exists in almost all living organisms and has important biological functions in a wide range of cellular activities [26]. In the present study, spermidine in ileal mucosa was increased by more than 6-fold in PC compared to NC, which possibly implicates an intestinal mucosal repair process. Of all the differential metabolites in this comparison, spermidine had the highest VIP score, further highlighting it as the most distinctive metabolic feature between PC and NC (Table 1 and Figure 2A). The metabolism of spermidine is tightly regulated [27]. Evidence suggests that spermidine is required for modulating the expression of genes of potassium ion channels, which are essential for the migration of epithelial cells during mucosal repair [28]. It was also reported in a rat model that increased mucosal spermidine concentration was associated with the repair of damaged mucosa [29].

Cell proliferation requires a supply of nucleotides for the associated increase in cellular processes such as DNA replication, mRNA production, and ribosomal RNA synthesis [30]. In the present study, ETEC infection also elevated cytidine by ~3.5 fold and altered metabolites (e.g., fructose 6-phosphate, gluconolactone, and ribose) involved in the pentose phosphate pathway in ileal mucosa, possibly indicating increased cell proliferation. Nucleotides can be produced through *de novo* synthesis or via the salvage pathway [31]. Nucleosides or purine and pyrimidine bases that are degraded from dietary nucleotides can be absorbed efficiently in the small intestine and used for nucleotide synthesis through the salvage pathway [32,33]. In rapidly proliferating cells such as enterocytes, the salvage pathway may be preferentially utilized [34]. Cytidine, being a pyrimidine nucleoside, can be directly used for the synthesis of cytidine monophosphate (CMP) or uridine monophosphate (UMP), which are ultimately phosphorylated to cytidine triphosphate (CTP) and uridine triphosphate (UTP). Therefore, the regulation of cytidine uptake and synthesis may play a role during the recovery stage of ETEC infection. However, the causes for the increase in cytidine but not the other nucleosides in the present study are yet to be answered. 

Several other metabolites that may indirectly relate to epithelial cell proliferation and differentiation were also increased in the ileal mucosa of ETEC challenged pigs including mannose, fructose, fructose-6-phosphate, glucose, and glucose-6-phosphate (Table 1). These metabolites are also involved in fructose and mannose degradation. In mammalian cells, mannose is an essential component of N-glycan, glycophospholipid, and glycoprotein [35,36]. The biosynthesis of N-glycan requires phosphorylated mannose 6-phosphate and mannose 1-phosphate that can be converted from fructose, fructose 6-phosphate, glucose, and glucose 6-phosphate via the mannose metabolic pathway [37,38]. Mannose *per se* can also enter the mannose metabolic pathway to produce N-glycan. However, the gastrointestinal tract of pigs cannot degrade plant polysaccharides containing homo- or hetero-polymers of mannose, free mannose, therefore, is produced almost entirely through intracellular N-glycan processing and degradation [39,40]. Recently, studies have revealed that regulation of compositional changes of N-glycosylation was related to intestinal epithelial cell growth and differentiation [41,42]. More specifically, compositional change of N-glycans (high degree of mannose) is responsive to the abundance of luminal nutrients and the presence of microbial products [41]. During intestinal epithelial cell differentiation, processing of oligomannose glycans was augmented and the diversity of N-glycans increased [42]. Glucose, glucose 6-phosphate, fructose, and fructose 6-phosphate are important initial substrates of glycolysis, which is one of the main metabolic pathways for energy production. The elevated fructose and glucose levels were also observed in the jejunal metabolites of pigs challenged with an ETEC F4 on d 6 PI [10].

Phospholipid metabolism such as the biosynthesis of phosphatidylcholine and the biosynthesis of phosphatidylethanolamine has been shown to be impacted by ETEC F18 infection. Levels of lipid metabolites such as lignoceric acid and pentadecanoic acid were found to be decreased in pigs from the positive control group when compared with pigs in the negative control. Phosphatidylcholine and phosphatidylethanolamine are the most abundant phospholipids in mammalian cells [43]. In the small intestine, phospholipids function to maintain cellular integrity of the enterocytes, regulate fatty acid uptake, and provide protection against harmful agents [43]. Alteration of phosphatidylcholine fatty acid composition and the depletion of phosphatidylcholine in mucus may weaken the mucus defense against luminal pathogens [44]. Lignoceric acid belongs to the family of saturated very long-chain fatty acid (VLCFA, chain length >20 carbons) with a chain length of 24 carbons, whereas pentadecanoic acid is a saturated long-chain fatty acid (LCFA, chain length >13 carbons) with a chain length of 15 carbons. Lignoceric acid is found in various tissues and synthesized through an elongation pathway and incorporated into sphingolipids [45]. Although the function of lignoceric acid in enteric infection and inflammation is not yet well understood, some evidence showed that the concentration of lignoceric acid in plasma and on the membrane of erythrocytes were significantly reduced in patients with inflammatory bowel disease (IBD) and Crohn’s disease (CD), and the concentration of pentadecanoic acid in the plasma of patients with IBD was also reduced [46,47]. Although data on intestinal morphology and tight junction proteins indicated that infected pigs were fully recovered from ETEC infection at 21 PI, the gene expression of inflammatory indicators (i.e., IL6, TNFA, and PTGS2) were still high in the ileal mucosa of ETEC infected pigs compared with non-infected pigs [5]. Increased epithelial cell proliferation may occur in response to different inflammatory stimuli. Therefore, decreased levels of lignoceric acid and pentadecanoic acid may indicate chronic inflammation in pigs from the positive control group. 

In summary, the differential metabolites in the ileal mucosa may suggest an ongoing intestinal mucosal repair process in ETEC F18-challenged pigs at d 21 PI after disease clearance. Furthermore, the repair of intestinal mucosa may be accompanied by increased activities of cell proliferation and differentiation, degradation and reconstruction of epithelial cell membrane-associated N-glycans, and alteration of the fatty acid composition of epithelial cell membranes. 

### 3.2. Metabolites Related to Microbial Metabolism

Pigs challenged with ETEC have been shown to have disrupted gut microbial homeostasis and altered profile of gut microbiota [13,48]. In the present study, changes in several differential metabolites such as sorbitol 6-phosphate, gluconic acid, and others may be modified by changes in gut microbiome. Sorbitol, or less commonly known as glucitol, is a sugar alcohol that can be found in many fruits and also in the kernels of corn [49]. Sorbitol is poorly absorbed by enterocytes in the small intestine, but can be utilized as a carbon source by the gut microbes such as bacteria in the phyla Proteobacteria and Firmicutes [50,51,52,53,54]. Briefly, sorbitol is transported and phosphorylated to sorbitol 6-phosphate, which is then further converted to fructose 6-phosphate and enters the glycolysis pathway [53]. Gluconic acid lactone and gluconic acid exist in natural sources such as plants, fruits, meats, and others by oxidation of glucose [55]. In bacteria, they are produced through a different glucose oxidation process catalyzed by glucose dehydrogenase [56]. Members of lactic acid bacteria such as *Lactobacillus* and *Bifidobacterium* are capable of utilizing gluconic acid [57,58]. 

In the present study, the level of conduritol-beta-epoxide was lower in the ileal mucosa of ETEC F18 challenged pigs. Conduritol-beta-epoxide is an effective beta-glucosidase inhibitor due to its high reactivity at the active site of the enzyme and its structural similarity with glucose [59]. Beta-glucosidases are a group of enzymes capable of hydrolyzing the glycosidic bond in various compounds such as cellobiose, oligosaccharides, glucosides, and glycoceramides, and thus play important roles in many metabolic processes including degradation of polysaccharides, breakdown of glycoprotein and glycolipid [60,61]. Although the literature has shown that conduritol-beta-epoxide can be found in some plants, little is known about the occurrence of conduritol and its epoxide in nature [62]. On the other hand, bacteria produce and secrete many beta-glucosidase inhibitors to suppress the growth of their competitors and gain advantage in obtaining available nutrients [60]. Decreased conduritol-beta-epoxide levels in ETEC F18 challenged pigs might be an indication that the gut microbial community has been disrupted. Thus, gut microbiome analysis across different gut segments would be considered to investigate the dynamic effects of ETEC infection on microbial diversity and relative population, and to investigate the interactions between host and microbiota metabolism.

In the colon digesta, differential metabolites such as 2-monoolein and maltose, which are end or intermediate products of digestion, were identified between the carbadox and probiotic groups. 2-Monoolein is a monoglyceride with one oleic acid chain bound to a glycerol molecule at the sn-2 position. It is the end product of triacylglycerol digestion by pancreatic lipase and can be absorbed in the small intestine [63]. Maltose is an intermediate metabolite in starch digestion, and can be further hydrolyzed by maltase at the brush boarder to glucose, which can be transported into enterocytes [64]. Decreased levels of these two metabolites in the colon digesta may indicate better lipid and carbohydrate digestion and absorption in the small intestine of pigs in the carbadox group compared to the probiotic group. Nutrients that escaped digestion or are the end product of digestion are constantly being utilized by gut microbiota, while exerting some beneficial effects for the host through microbial metabolites [65]. Lactic acid bacteria may benefit from increased levels of maltose because of their capability to utilize maltose through the maltose and trehalose catabolic pathways [66]. In the current study, lactic acid was also identified as a differential metabolite that was increased in pigs in the probiotic group in comparison to the carbadox group. Lactic acid is produced by lactic acid bacteria and may benefit the host by inhibiting the growth of enteropathogens [67]. Lactic acid can be further converted to short-chain fatty acids including butyric acid and propionic acid, which can be used by intestinal epithelial cells as a source of energy [65,68,69]. Several other studies have also reported an increase in the lactic acid bacteria population with the supplementation of *Bacillus* probiotics [20,70,71]. Current findings in colonic metabolites may demonstrate the differences in modes of action between carbadox and probiotics in promoting gut health. 

## 4. Material and Methods

### 4.1. Ethical Statement

The protocol for this experiment was reviewed and approved by the Institutional Animal Care and Use Committee (IACUC # 19322) of the University of California, Davis. 

### 4.2. Animals, Housing and Experimental Design

A total of 48 weanling pigs (21 d old; 6.17 ± 0.36 kg) with an equal number of barrows and gilts were used in this experiment. The sows and piglets used in this experiment did not receive *E. coli* vaccines, antibiotic injections, or antibiotics in creep feed. Before weaning, fecal samples were collected from sows and all their piglets destined for this experiment to verify the absence of β-hemolytic *E. coli*. The *E. coli* F18 fimbriae receptor status in all piglets were also tested based on the methods described previously in Kreuzer et al. [72]. All pigs used in this experiment were susceptible to *E. coli* F18 infection and free of *E. coli* F18 prior to the experiment. After weaning, all pigs were transferred to the Cole facility at the University of California, Davis, and were housed in individual pens (0.61 m × 1.22 m) for 28 days including seven days before and 21 days after the first *E. coli* challenge. All pigs had free access to feed and water. Animal rooms were equipped with fans and heaters to achieve the desired thermoneutral zone for nursery pigs. The light period was provided for 12 h starting from 0730 h.

Pigs were given a seven day adaption period and on d 7 or d 0 post-inoculation (PI), all pigs in the *E. coli* challenge treatments were orally inoculated with an *E. coli* F18. Pigs were then challenged again with one dose of *E. coli* F18 on d 1 and d 2 PI for a total of three consecutive days. The *E. coli* F18 were isolated from a field disease outbreak by the University of Illinois Veterinary Diagnostic Lab (isolate number: U.IL-VDL # 05-27242). The *E. coli* F18 were provided at 10^10^ CFU per 3-mL dose in phosphate-buffered saline and expresses heat-labile toxin, heat-stable toxin b, and Shiga-like toxins. This dose causes mild diarrhea based on our previous research [4,16].

Pigs were randomly assigned to one of four treatments in a randomized complete block design with weight within sex and litter as the blocks and individual pig as the experimental unit. There were 12 replicates per treatment. The treatments included: (1) negative control (NC), control diet without *E. coli* challenge, (2) positive control (PC), control diet with E. coli challenge, (3) antibiotic growth promotor (AGP), control diet supplemented with carbadox at 50 mg/kg and with E. coli challenge, and (4) probiotics (PRO), control diet supplemented with a *Bacillus subtilis* sp. at 2.59 × 10^9^ CFU/kg diet and with *E. coli* challenge. All diets were based on corn, dried whey, soybean meal, and fish meal, and met the current estimates for the nutrient requirements of nursery pigs (Table 3) [73]. The experimental diets were fed to pigs as a 2-phase feeding program with weeks 1 and 2 as phase 1 and weeks 3 and 4 as phase 2. Spray-dried plasma, antibiotics, and high levels of zinc oxide exceeding recommendation were not included in the diets. Growth performance, clinical data, and several blood parameters were reported in our previously published research [5]. No block effects were observed in blood parameters. 

### 4.3. Sample Collection

All pigs were euthanized at the end of the experiment (d 21 PI). Before euthanasia, pigs were anesthetized with a 1 mL mixture of 100 mg telazol, 50 mg ketamine, and 50 mg xylazine (2:1:1) by intramuscular injection. After anesthesia, intracardiac injection with 78 mg sodium pentobarbital (Vortech Pharmaceuticals, Ltd., Dearborn, MI, USA) per 1 kg of BW was used to euthanize each pig. Ileum mucosa and colon digesta were collected from all pigs and immediately snap-frozen in liquid nitrogen for untargeted metabolomics analysis. 

### 4.4. Untargeted Metabolomics Analysis

The untargeted metabolomics analysis was performed by the NIH West Coast Metabolomics Center using gas chromatography (Agilent 6890 gas chromatograph controlled using Leco ChromaTOF software version 2.32) coupled with time-of-flight mass spectrometry (GC/TOF-MS) (Leco Pegasus IV time-of flight mass spectrometer controlled using Leco ChromaTOF software version 2.32). Metabolite extraction was performed following the procedures described in a previously published article [74]. Briefly, frozen samples (approximately 10 mg) were homogenized using a Retsch ball mill for 30 s at 25 times/s. After homogenization, a prechilled (−20 °C) extraction solution (isopropanol/acetonitrile/water at the volume ratio 3:3:2, degassed with liquid nitrogen) was added at a volume of 1 mL solution/20 mg of sample. Samples were then vortexed and shaken for metabolite extraction. After centrifugation at 12,800× *g* for 2 min, the supernatant was collected and separated into two equal aliquots and concentrated at room temperature for 4 h in a cold-trap vacuum concentrator (Labconco Centrivap). To separate complex lipids and waxes, the residue was re-suspended in 500 µL 50% aqueous acetonitrile, and centrifuged at 12,800× *g* for 2 min. The resultant supernatant was collected and concentrated in the vacuum concentrator. Dried sample extracts were derivatized and mixed with internal retention index markers (fatty acid methyl esters with chain length of C8 to C30). The sample was injected for GC/TOF analysis, and all samples were analyzed in a single batch [74]. 

The raw data were directly preprocessed using Leco ChromaTOF software (v.2.32) for automatic mass spectral deconvolution and peak detection at signal/noise levels of 5:1. The spectral data were further refined using the BinBase algorithm [75]. All metabolite spectra in BinBase were matched against the Fiehn mass spectral library and the NIST spectral library based on the retention index, validation of unique ions and apex masses, and mass spectrum similarity. The identified metabolites were reported for compound names with external database identifiers (InChI key, PubChem ID and KEGG ID) and signal intensity of peak height (mz value, mass-to-charge ratio). 

### 4.5. Data Analysis

The metabolomics data were analyzed using different modules of a web-based platform MetaboAnalyst (https://www.metaboanalyst.ca). Data were normalized using logarithmic transformation and auto-scaling. Fold change analysis and *t*-tests were conducted to determine the fold change and significance of each identified metabolite. Statistical significance was declared at false discovery rate (FDR, Benjamini and Hochberg correction) adjusted *p* ≤ 0.05. Partial least squares discriminant analysis (PLS-DA) was carried out to further explore the differential metabolites among the treatment groups. Pathway analysis and metabolite set enrichment analysis were performed on metabolites that had a Variable Importance in Projection (VIP) score > 1 [76,77]. The following criteria were used to identify the specific compounds responsible for the differences: VIP > 1, fold change > 2, and FDR < 0.2.

## 5. Conclusions

In conclusion, our study identified several differential metabolites and metabolic pathways in the ileal mucosa of ETEC F18 challenged pigs on d 21 PI. These differentially expressed metabolites and impacted metabolic pathways between pigs from the negative control and positive control groups may be associated with the recovery of ETEC challenged diarrheal pigs on d 21 PI. More research will be needed to target several remarkably changed metabolites in the small intestine to further decipher their impacts on the gut health of pigs. Future studies with targeted metabolomics that are hypothesis-driven and have a high level of precision may provide more insights into host metabolic changes in response to ETEC challenge and in recovery. The exploration of host and gut microbiome interaction will be important to unravel the mechanisms of ETEC infection and to discover effective nutritional strategies. 

## Figures and Tables

**Table 1:** Ileal mucosa metabolites that differed among the dietary treatment groups.

Metabolites	Fold Change ^1^	VIP ^2^	FDR ^3^
positive control vs. negative control			
spermidine	6.25	1.87	0.010
cytidine	3.45	1.65	0.030
gluconic acid	3.23	1.63	0.030
gluconic acid lactone	3.13	1.81	0.010
gulonic acid	2.94	1.43	0.057
fructose	2.94	1.51	0.044
fructose-6-phosphate	2.33	1.34	0.080
mannose	2.27	1.44	0.055
sorbitol-6-phosphate	2.17	1.22	0.104
glucose	2.04	1.30	0.086
glucose-6-phosphate	2.04	1.21	0.105
adipic acid	0.44	1.36	0.076
pentadecanoic acid	0.43	1.52	0.044
lignoceric acid	0.41	1.26	0.093
glutaric acid	0.38	1.55	0.044
pyrophosphate	0.38	1.17	0.118
conduritol-beta-epoxide	0.26	1.47	0.049
adenosine-5-monophosphate	0.20	1.53	0.044
succinic acid	0.17	1.44	0.055
carbadox vs. probiotics			
1-monoolein	0.50	2.64	0.052

^1^ Fold change values less than one indicate that the differential metabolites were reduced in the positive control compared to the negative control or reduced in carbadox compared to probiotics. ^2^ VIP, variable importance in the projection. ^3^ False discovery rate.

**Table 2:** Colonic digesta metabolites that differed among dietary treatment groups.

Metabolites	Fold Change ^1^	VIP ^2^	FDR ^3^
negative control vs. positive control			
adenine	3.63	2.40	0.105
positive control vs. carbadox			
phosphate	0.49	2.79	0.085
carbadox vs. probiotics			
2-monoolein	0.28	1.92	0.187
lactic acid	0.37	2.15	0.138
maltose	0.37	2.12	0.138
adenine	0.42	1.93	0.187
aspartic acid	0.50	1.92	0.187
phosphate	2.03	2.44	0.054

^1^ Fold change values less than one indicate that the differential metabolites are reduced in former treatment group compared to the latter treatment group. ^2^ VIP, variable importance in the projection. ^3^ False discovery rate.

**Table 3:** Ingredient compositions of experimental diets ^1^.

Ingredient, %	Control, Phase I	Control, Phase II
Corn	44.41	57.27
Dried whey	15.00	10.00
Soybean meal	18.00	22.00
Fish meal	10.00	7.00
Lactose	6.00	-
Soy protein concentrate	3.00	-
Soybean oil	2.00	2.00
Limestone	0.56	0.70
L-Lysine·HCl	0.21	0.23
DL-Methionine	0.08	0.05
L-Threonine	0.04	0.05
Salt	0.40	0.40
Vit-mineral, Sow 6 ^2^	0.30	0.30
Total	100.00	100.00
Calculated energy and nutrient		
Metabolizable energy, kcal/kg	3463	3429
Net energy, kcal/kg	2601	2575
Crude protein, %	22.27	20.80
Arg,^3^ %	1.23	1.15
His,^3^ %	0.49	0.47
Ile,^3^ %	0.83	0.76
Leu,^3^ %	1.62	1.55
Lys,^3^ %	1.35	1.23
Met,^3^ %	0.45	0.39
Thr,^3^ %	0.79	0.73
Trp,^3^ %	0.23	0.21
Val,^3^ %	0.91	0.84
Met + Cys,^3^ %	0.74	0.68
Phe + Tyr,^3^ %	1.45	1.38
Ca, %	0.80	0.70
Total P, %	0.68	0.59
Digestible P, %	0.47	0.37
Analyzed nutrient, as-is		
Dry matter, %	90.70	89.90
Crude protein, %	23.13	21.30
ADF, %	7.26	9.35
NDF, %	2.54	3.60
Ca, %	0.96	0.88
P, %	0.71	0.59

^1^ In each phase, two additional diets were formulated by adding probiotics or carbadox to the control diet, respectively. The dose for probiotics was 500 mg/kg, which was equal to 2.56 × 109 CFU/kg diet. The dose for carbadox was 50 mg/kg diet. ^2^ Provided the following quantities of vitamins and micro minerals per kilogram of complete diet: Vitamin A as retinyl acetate, 11,136 IU; vitamin D3 as cholecalciferol, 2208 IU; vitamin E as DL-alpha tocopheryl acetate, 66 IU; vitamin K as menadione dimethylprimidinol bisulfite, 1.42 mg; thiamin as thiamine mononitrate, 0.24 mg; riboflavin, 6.59 mg; pyridoxine as pyridoxine hydrochloride, 0.24 mg; vitamin B12, 0.03 mg; D-pantothenic acid as D-calcium pantothenate, 23.5 mg; niacin, 44.1 mg; folic acid, 1.59 mg; biotin, 0.44 mg; Cu, 20 mg as copper sulfate and copper chloride; Fe, 126 mg as ferrous sulfate; I, 1.26 mg as ethylenediamine dihydriodide; Mn, 60.2 mg as manganese sulfate; Se, 0.3 mg as sodium selenite and selenium yeast; and Zn, 125.1 mg as zinc sulfate. ^3^ Amino acids were indicated as standardized ileal digestible AA.

## Data Availability

The data presented in this study are available on request from the corresponding author. The data are not publicly available due to the authors may process more data analysis with the raw data.
